# Association between serum lactate trajectories and short-term mortality in pediatric acute kidney injury patients

**DOI:** 10.1016/j.jped.2026.101603

**Published:** 2026-09-06

**Authors:** Xiaodong Jiang, Yu Zhang, Hua Lu

**Affiliations:** a Department of Pediatrics, Affiliated Hospital of Zunyi Medical University, Zunyi, Guizhou, China; b Department of Pediatrics, Guizhou Children's Hospital, Zunyi, Guizhou, China; c Department of Hospital Infection Control, Chongqing Mental Health Center, Chongqing, China; d Department of Pediatrics, Shapingba Hospital affiliated to Chongqing University (Shapingba District People's Hospital of Chongqing), No. 2, Jialang Road, Shapingba District, Chongqing, China

**Keywords:** Lactates, Acute kidney injury, Mortality, Critical care, Trajectory analysis, Pediatrics

## Abstract

**Objective:**

Acute kidney injury (AKI) is a life-threatening condition in children. Serum lactate is a critical indicator of oxygen metabolism and tissue hypoperfusion. This study investigated the association between early dynamic lactate trajectories during pediatric intensive care unit (PICU) and 30-day mortality in pediatric patients with AKI.

**Methods:**

This single-center retrospective study included 1056 critically ill children with AKI admitted to the PICU. Multivariable Cox regression and restricted cubic spline (RCS) models were used to evaluate the association between admission lactate levels and 30-day mortality. Latent growth mixture model (LGMM) was used to identify distinct lactate trajectories based on daily measurements during the first four days of admission. Multivariable Cox regression and Kaplan-Meier analysis were then conducted to assess the association between trajectory classes and 30-day mortality.

**Results:**

Admission lactate was independently associated with 30-day mortality (HR 1.13, 95% CI 1.08–1.18, p < 0.01), with a monotonic dose-response relationship (p for non-linearity = 0.46). Three distinct lactate trajectories were identified: Class 1 (high decreasing group, 5.11%), Class 2 (low stable group, 92.05%), and Class 3 (persistent high group, 2.84%). Compared with Class 2, both Class 1 (HR 3.37, 95% CI 1.83–6.24, p < 0.01) and Class 3 (HR 6.24, 95% CI 2.95–13.17, p < 0.01) were associated with significantly increased mortality. Kaplan-Meier curves confirmed the poorest survival in Class 3.

**Conclusion:**

Both admission lactate level and early dynamic lactate trajectory provide valuable prognostic information in pediatric AKI patients. Identifying lactate patterns facilitates early risk stratification and guides individualized management.

## Introduction

Acute kidney injury (AKI) is defined as an abrupt decrease in kidney function, typically diagnosed by an increase in serum creatinine or a decrease in urine output, according to the Kidney Disease: Improving Global Outcomes (KDIGO) criteria. AKI is a common complication in critically ill patients, affecting up to 25% of children in the intensive care unit (ICU) [1,2]. Importantly, AKI is a leading cause of in-hospital morbidity and mortality. A recent meta-analysis of 133,876 hospitalized children with AKI reported a pooled in-hospital mortality rate of 18.27%, with mortality increasing progressively from 8.19% in stage 1 to 27.78% in stage 3 [3]. Beyond its impact on patient outcomes, AKI imposes a substantial economic burden on healthcare systems [4,5]. These findings underscore the urgent need for early identification and risk stratification of high-risk pediatric AKI patients to improve clinical outcomes and reduce healthcare expenditures. Although various critical illness scoring tools and single biomarkers are currently used to assess the prognosis of children with AKI, they have notable limitations in clinical practice. First, most scoring systems are based on data collected within the first 24 h of admission and cannot dynamically reflect real time changes in a patient's condition. Second, traditional indicators such as creatinine and urine output respond slowly to renal injury, making early warning difficult. Therefore, there is an urgent need for novel prognostic markers that can capture dynamic disease progression and enable continuous bedside monitoring, thereby facilitating early identification and timely intervention in high-risk pediatric AKI patients.

Lactate is a metabolite derived from pyruvate under hypoxic or anaerobic conditions and serves as a sensitive marker of tissue hypoperfusion and cellular hypoxia. It has been widely used for risk assessment and prognosis prediction in critically ill patients [6]. In populations with sepsis, pneumonia, trauma, or post-cardiac surgery, both admission lactate levels and lactate clearance have been shown to be closely associated with mortality [7, 8, 9, 10, 11]. In recent years, the prognostic value of lactate in patients with AKI has gained increasing attention. Multiple studies have demonstrated that elevated admission lactate is significantly associated with increased in-hospital mortality in AKI patients [12, 13, 14, 15]. However, most existing studies have relied on lactate measurements at a single time point. In fact, the clinical course of critically ill patients is continuously evolving, and therapeutic interventions constantly influence lactate production and clearance. Consequently, a single lactate value may not adequately capture disease progression or response to treatment, potentially leading to underestimation or overestimation of mortality risk. Trajectory analysis has been applied in adult populations with AKI and other critical conditions, revealing distinct lactate patterns that are strongly associated with prognosis. However, evidence regarding the association between dynamic lactate trajectories and short-term outcomes in children with AKI remains lacking. Therefore, this study aims to identify distinct lactate trajectories based on serial measurements over the first four days of PICU admission using latent growth mixture model and to examine their association with 30-day mortality in pediatric AKI patients. The findings may provide a novel dynamic marker for early risk stratification and targeted intervention in this vulnerable population.

## Methods

### Data source and study population

**Figure 1:**
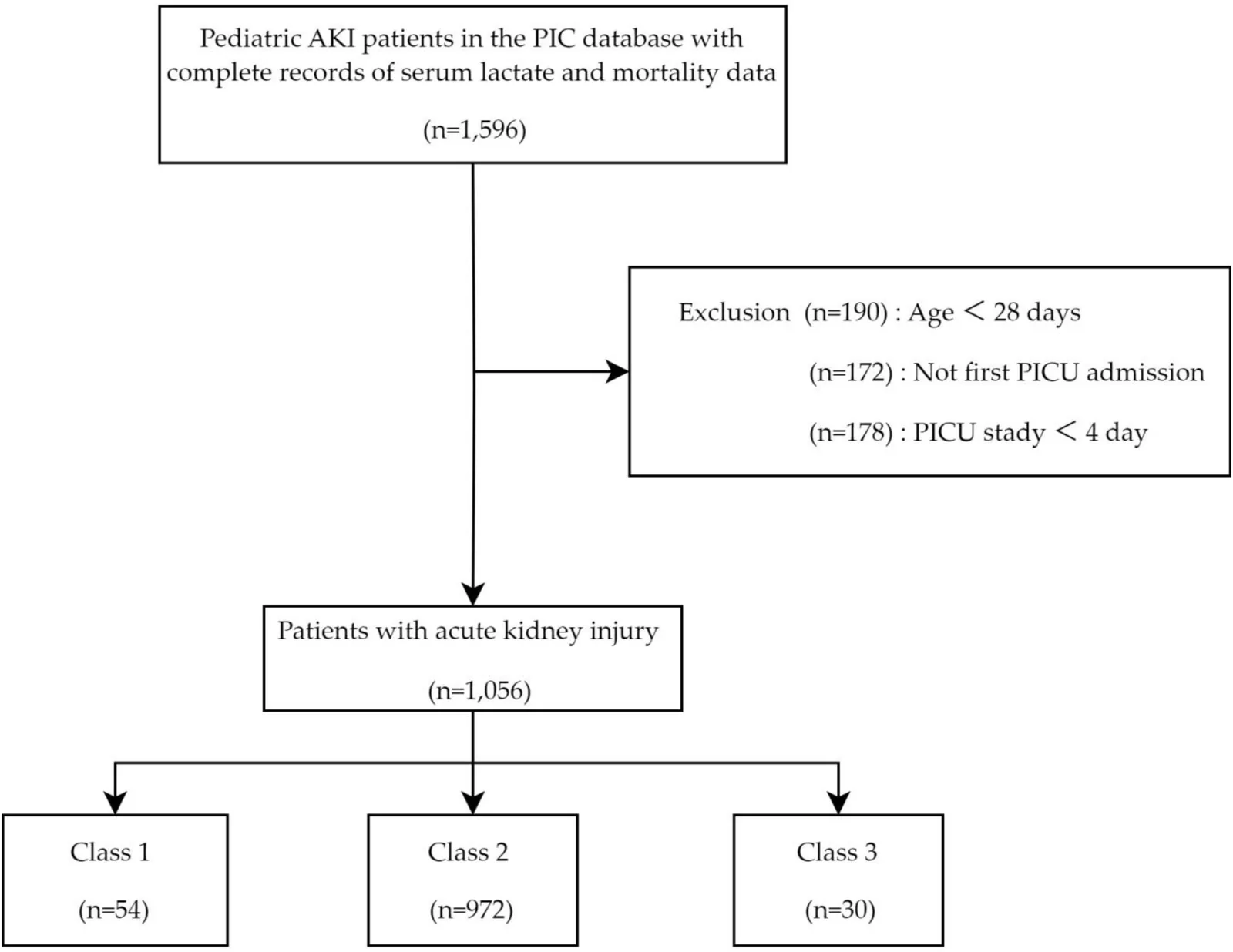
The flowchart of this study.

The study utilized data from the Pediatric Intensive Care (PIC) database (version 1.1.0), a comprehensive Chinese pediatric critical care repository containing detailed clinical records of patients admitted to the Children’s Hospital of Zhejiang University School of Medicine between 2010 and 2018. The inclusion criteria for the patients in this study were as follows: (1) aged > 28 days and < 18 years; (2) admission to the pediatric intensive care unit (PICU) with a diagnosis of AKI, as defined by the Kidney Disease: Improving Global Outcomes (KDIGO) criteria; (3) complete daily measurement of serum lactate concentration on each of the first 4 days after PICU admission. AKI staging was determined according to the KDIGO criteria, based on both serum creatinine (SCr) and urine output: Stage 1 was defined as an increase in SCr ≥ 26.5 μmol/L or 1.5–1.9 times baseline, or urine output < 0.5 mL/(kg·h) for 6–12 h; Stage 2 as SCr 2–2.9 times baseline or urine output < 0.5 mL/(kg·h) for ≥ 12 h; and Stage 3 as SCr ≥ 3 times baseline, SCr ≥ 354 μmol/L, urine output < 0.3 mL/(kg·h) for ≥ 24 h, or anuria for ≥ 12 h. When the serum creatinine and urine output criteria indicated different AKI stages, the more severe stage was assigned as the final stage. Baseline serum creatinine was defined as the lowest creatinine value recorded within the 7 days prior to PICU admission. Patients were excluded if they met any of the following criteria: (1) neonates ≤ 28 days old; (2) readmissions to the PICU; (3) PICU stays shorter than 4 days. A total of 1056 patients were included in the final analysis. The patient selection process is illustrated in Figure 1.

### Data collection

Clinically relevant variables were extracted from the database using structured query language (SQL). The selected covariates encompassed demographics, vital signs, comorbidities, laboratory results, and treatments. Continuous clinical variables (e.g., vital signs and laboratory results) were collected as the most extreme value within the initial 24 h of PICU admission. For analysis of serum lactate trajectories, this study extracted the maximum daily lactate value for each patient over four consecutive days following PICU admission. The diagnosis of comorbid conditions was based on ICD-10 classifications. The primary outcome of this study was 30-day all-cause mortality among patients with AKI, defined as death occurring following 30 days of PICU admission. To reduce the impact of potential bias, the study excluded variables with a missing rate of >20%. For data with a missing rate of <20%, we used the ‘mice’ package of the R software and applied the random forest method of multiple imputation.

### Statistical analysis

R software (version 4.4.2, R Foundation for Statistical Computing, Vienna, Austria) was utilized for statistical analysis. Latent growth mixture model (LGMM) accounts for population heterogeneity by modeling distinct sub-populations, each represented by unique mean trajectory curves [16]. Models comprising 2 to 6 latent classes were developed, with the optimal number of classes selected based on model fit indices, including the Akaike Information Criterion (AIC), Bayesian Information Criterion (BIC), sample-size adjusted BIC (SABIC), and entropy values. Lower AIC, BIC, and SABIC values indicate better model fit. Entropy, ranging from 0 to 1, was used to assess classification certainty, with values > 0.7 indicating acceptable discrimination. To ensure model stability and clinical interpretability, the study required that the sample size of each latent class exceed 1% of the total study population. In addition, the average posterior probability for class membership was required to be ≥ 70% for all identified classes.

After patient stratification, baseline characteristics were compared across the groups. Normally distributed continuous variables are presented as mean ± standard deviation and were compared using analysis of variance (ANOVA). Non-normally distributed continuous variables are presented as median with interquartile range (25th percentile, 75th percentile) and were compared using the Kruskal–Wallis test. Categorical variables are expressed as frequencies (n) and percentages (%) and were compared using the chi-square test. A multivariable Cox proportional hazards model was used to assess the associations of admission serum lactate level and different lactate trajectory patterns with 30-day mortality, respectively. To avoid multicollinearity, variables with a variance inflation factor (VIF) greater than 5 were excluded. To explore potential nonlinear relationships between admission lactate level and 30-day mortality, this study employed restricted cubic splines (RCS) within a Cox proportional hazards model. The likelihood ratio test was used to compare the nonlinear model with a linear model. Kaplan–Meier survival analysis was performed to evaluate endpoint incidence across different lactate trajectory groups. All statistical tests were two-sided, and a *p*-value < 0.05 was considered statistically significant.

## Results

### Lactate trajectory classification

**Figure 2:**
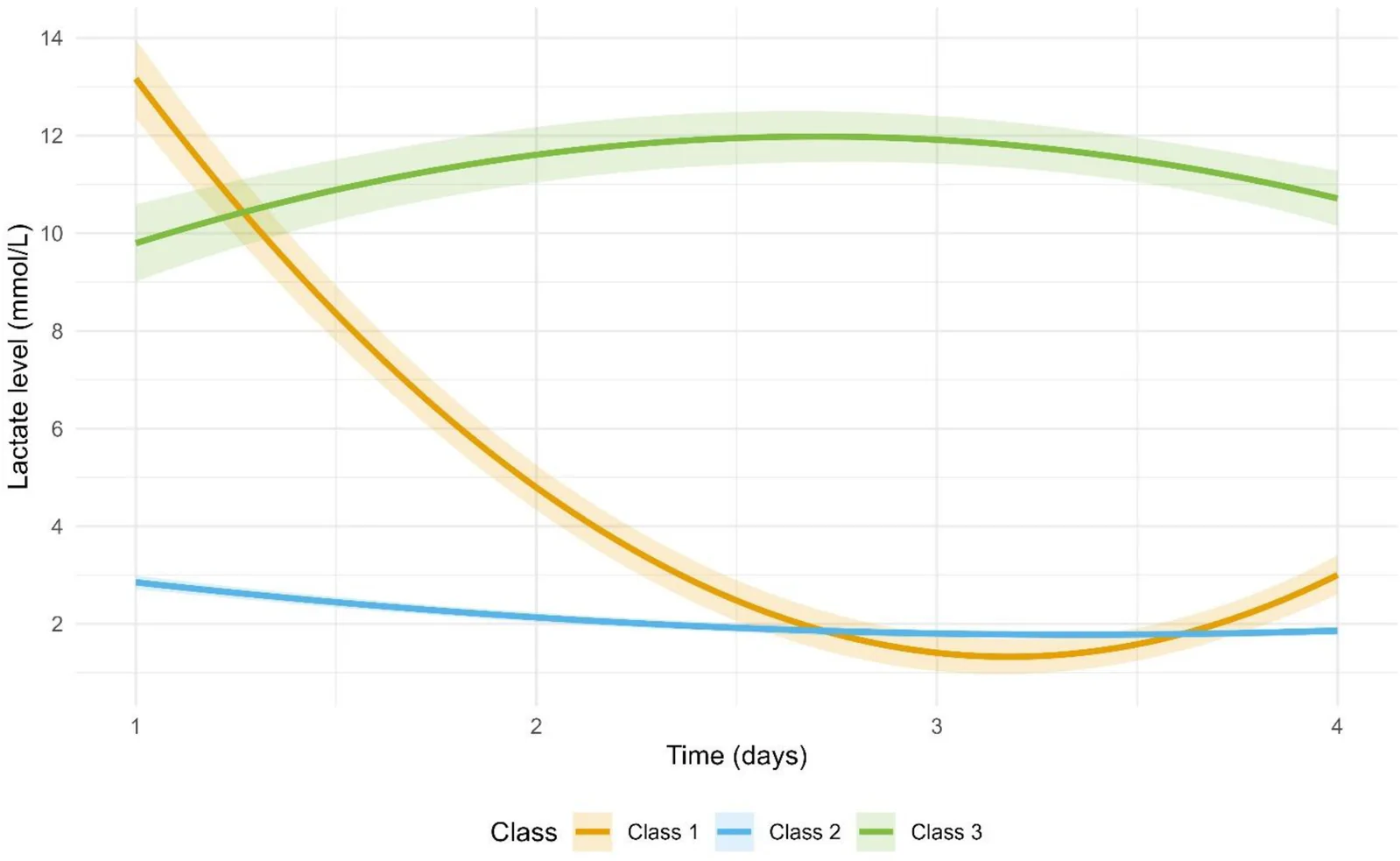
Lactate-based trajectories for pediatric patients with AKI.

A total of 1056 patients diagnosed with AKI were included in the final analysis. Table 1 presents a comparison of lactate trajectory models fitted using LGMM. Models ranging from two to six classes were evaluated. The three-class model demonstrated the optimal overall performance based on BIC (16678.02), AIC (16603.59), Entropy (0.985), and balanced class proportions. The two-class model showed limited discriminatory ability, while models with four or more classes suffered from small sample sizes in some subgroups and yielded near-empty classes. The posterior probabilities for trajectory classes 1 to 3 were 95.42%, 99.53%, 98.80%, respectively, indicating that the model had acceptable accuracy. Therefore, the three-class model was selected for subsequent analyses, as it provided the best balance between statistical fit and clinical interpretability. As shown in Figure 2, the three lactate trajectory classes over the first 96 h of PICU stay were: Class 1 (High decreasing group, n = 54, 5.11%), in which patients had markedly elevated admission lactate but showed a rapid decline within 96 h; Class 2 (Low stable group, n = 972, 92.05%), in which patients maintained near-normal lactate levels throughout the first 4 days; and Class 3 (Persistent high group, n = 30, 2.84%), in which patients exhibited persistently elevated lactate levels despite treatment. Shaded areas represent the 95% confidence intervals for each trajectory class.

**Table 1:** Model fit indices for latent growth mixture models with 2–6 classes.

Number of classes	Log Likelihood	AIC	BIC	SABIC	Entropy	%Class 1	%Class 2	%Class 3	%Class 4	%Class 5	%Class 6
1	−9164.286	18342.57	18377.31	18355.08	1	100					
2	−8687.652	17397.30	17451.89	17416.95	0.9780444	6.155303	93.8447				
3	−8286.793	16603.59	16678.02	16630.38	0.9851330	5.113636	92.04545	2.840909			
4	−8284.208	16606.42	16700.70	16640.35	0.8221394	5.113636	0	92.234848	2.651515		
5	−8286.793	16619.59	16733.72	16660.67	0.6439838	5.303030	0	91.856061	0	2.840909	
6	−8284.208	16622.42	16756.40	16670.64	0.7041831	5.113636	0	0	0	92.234848	2.651515

Abbreviations: AIC, Akaike information criterion, BIC, Bayesian information criteria, SABIC, sample-adjusted information criteria.

### Clinical characteristics

Table 2 presents the baseline characteristics of the study cohort. Data from 1056 patients with AKI were analyzed, with a mean age of 1.38 years. The overall 30-day mortality rate was 9.47%. Statistically significant differences were observed across three groups regarding heart rate, temperature, BUN, creatinine, potassium, CKMB, pH, lactate, potassium, PT, INR, APTT, ALT, and AST.

**Table 2:** Baseline clinical characteristics of pediatric patients with AKI stratified by lactate trajectory groups.

Variables	Overall (n = 1056)	Class 1 (n = 54)	Class 2 (n = 972)	Class 3 (n = 30)	*p*-Value
**Demographics**					
Age, years	1.38(0.45,5.00)	1.09(0.36,4.33)	1.41(0.45,5.00)	1.28(0.50,5.29)	0.73
Gender (*n*, %)					0.40
Female	443(41.95)	23(42.59)	411(42.28)	9(30.00)	
Male	613(58.05)	31(57.41)	561(57.72)	21(70.00)	
**Comorbidities**					
Sepsis, (n, %)	28(2.66)	2(3.70)	25(2.58)	1(3.33)	0.86
Shock, (n, %)	7(0.66)	0(0.00)	6(0.62)	1(3.33)	0.16
Malignant tumor, (n, %)	54(5.12)	3(5.56)	48(4.95)	3(10.00)	0.46
Hepatic dysfunction, (n, %)	27(2.56)	2(3.70)	22(2.27)	3(10.00)	0.03
AKI stage, (n, %)					0.47
Stage 1	557(52.75)	27(50.00)	519(53.40)	11(36.67)	
Stage 2	1(0.09)	0(0.00)	1(0.10)	0(0.00)	
Stage 3	498(47.16)	27(50.00)	452(46.50)	19(63.33)	
**Vital signs**					
Heart rate, bpm	140.93 ± 27.20	151.56 ± 26.58	140.50 ± 27.03	135.73 ± 30.35	<0.01
Respiratory rate, bpm	37.30 ± 13.61	38.63 ± 14.25	37.16 ± 13.57	39.30 ± 13.90	0.53
Temperature, °C	37.48 ± 1.18	36.92 ± 1.61	37.50 ± 1.14	37.53 ± 1.48	<0.01
**Laboratory results**					
RBC, 10^12^/L	4.02 ± 0.86	3.93 ± 1.15	4.02 ± 0.84	4.12 ± 1.00	0.71
WBC, 10^9^/L	15.21 ± 34.49	15.78 ± 8.45	15.31 ± 35.87	11.06 ± 6.99	0.80
Platelets, 10^9^/L	247.34 ± 156.10	257.78 ± 166.32	246.22 ± 154.66	264.90 ± 185.67	0.72
Hemoglobin, g/L	118.58 ± 24.28	118.35 ± 29.63	118.60 ± 24.01	118.27 ± 22.93	0.99
PCT, ng/mL	0.23 ± 0.14	0.24 ± 0.15	0.23 ± 0.14	0.25 ± 0.17	0.73
CRP, mg/L	26.00(6.00,62.96)	18.50(5.00,44.50)	27.00(6.00,64.00)	10.00(4.00,36.50)	0.05
ALT, U/L	32.00(20.00,64.00)	119.50(49.75,379.50)	31.00(19.00,54.00)	92.00(35.00,257.50)	<0.01
AST, U/L	79.00(37.75,151.00)	227.00(100.75,520.25)	74.00(36.00,140.00)	124.00(53.50,402.75)	<0.01
BUN, mmol/L	4.32(3.04,6.00)	6.08(4.91,8.01)	4.19(3.02,5.86)	3.92(2.43,6.82)	<0.01
Creatinine, μmol/L	47.00(39.00,59.08)	68.15(56.00,87.75)	46.00(38.85,57.00)	54.70(41.05,79.48)	<0.01
Albumin, g/L	36.31 ± 7.19	35.05 ± 7.39	36.42 ± 7.18	35.01 ± 7.03	0.24
CKMB, U/L	42.00(24.00,72.00)	108.00(44.75,274.00)	41.00(23.00,66.25)	40.00(25.25,145.50)	<0.01
pH	7.32(7.26,7.38)	7.12(7.04,7.23)	7.33(7.27,7.39)	7.29(7.19,7.35)	<0.01
Lactate, mmol/L	3.00(1.90,4.80)	12.75(10.80,16.00)	2.80(1.80,4.30)	9.40(4.33,12.60)	<0.01
Potassium, mmol/L	4.20(3.80,4.70)	4.90(3.83,5.88)	4.20(3.80,4.60)	4.45(4.10,4.88)	<0.01
Sodium, mmol/L	133.00(130.00,136.00)	133.00(129.25,137.00)	133.00(130.00,136.00)	133.00(131.00,135.75)	0.88
PT, s	14.55(12.50,17.63)	20.25(15.63,28.25)	14.40(12.40,17.20)	16.65(13.65,22.63)	<0.01
APTT, s	39.70(30.48,56.58)	54.75(42.58,93.05)	39.10(30.10,54.25)	46.40(33.20,88.58)	<0.01
INR	1.22(1.04,1.48)	1.64(1.31,2.30)	1.20(1.03,1.44)	1.35(1.10,1.77)	<0.01
**Interventions**					
Vasoactive drug, (n, %)	294(27.84)	13(24.07)	270(27.78)	11(36.67)	0.46
**Outcome**					
30-day mortality, (n, %)	100(9.47)	17(31.48)	72(7.41)	11(36.67)	<0.01

Abbreviations: WBC, white blood cells; RBC, red blood cells; PCT, Procalcitonin; CRP, C-reactive protein; BUN, blood urea nitrogen; CKMB, Creatine kinase-MB isoenzyme; PT, Prothrombin time; INR, international normalized ratio; APTT, Activated partial thromboplastin time; ALT, alanine aminotransferase; AST, aspartate aminotransferase.

### Association between serum lactate and risk of 30-day mortality in patients with AKI

**Figure 3:**
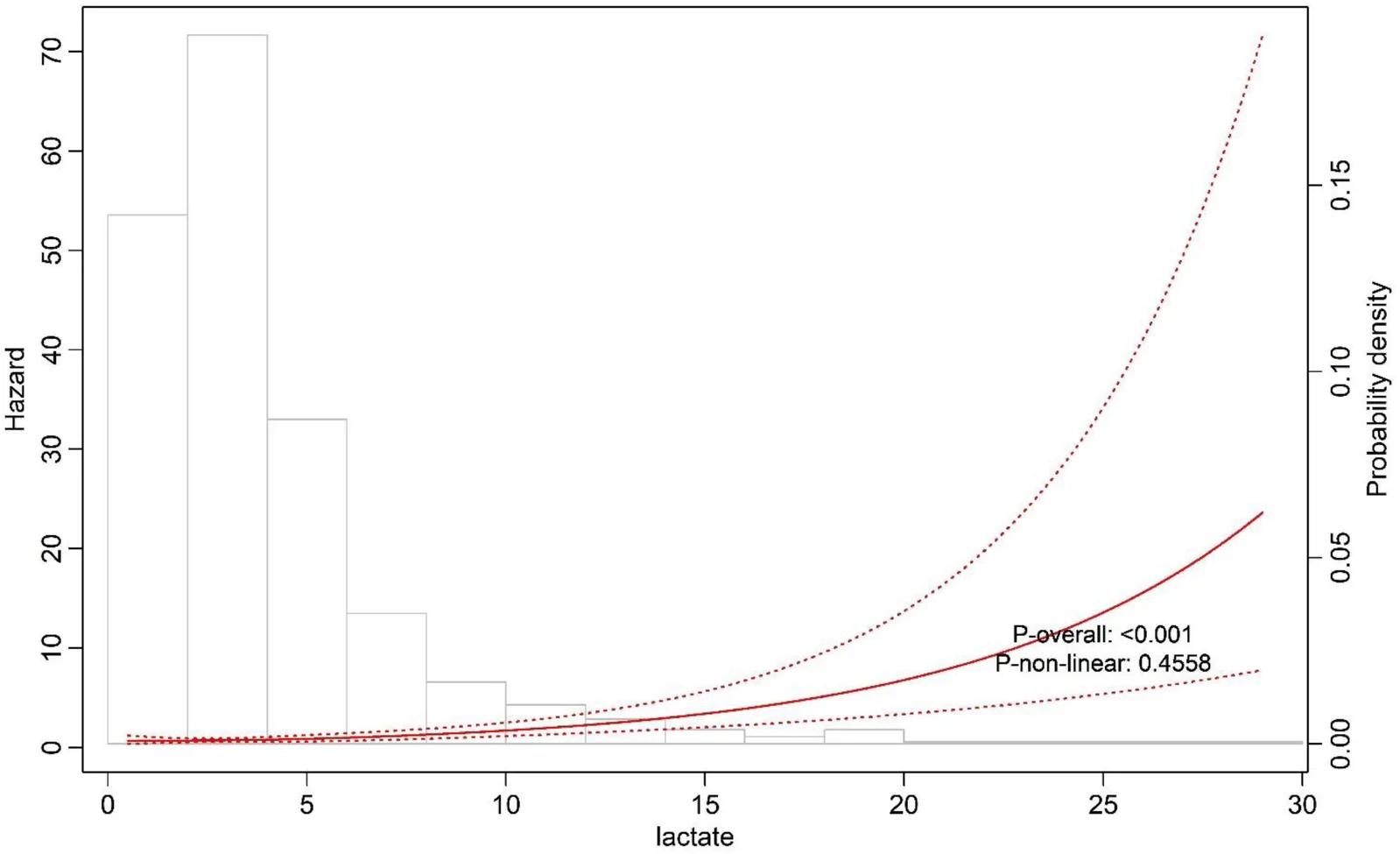
Restricted cubic spline analyses of the association of admission lactate with all cause death in 30 days.

**Figure 4:**
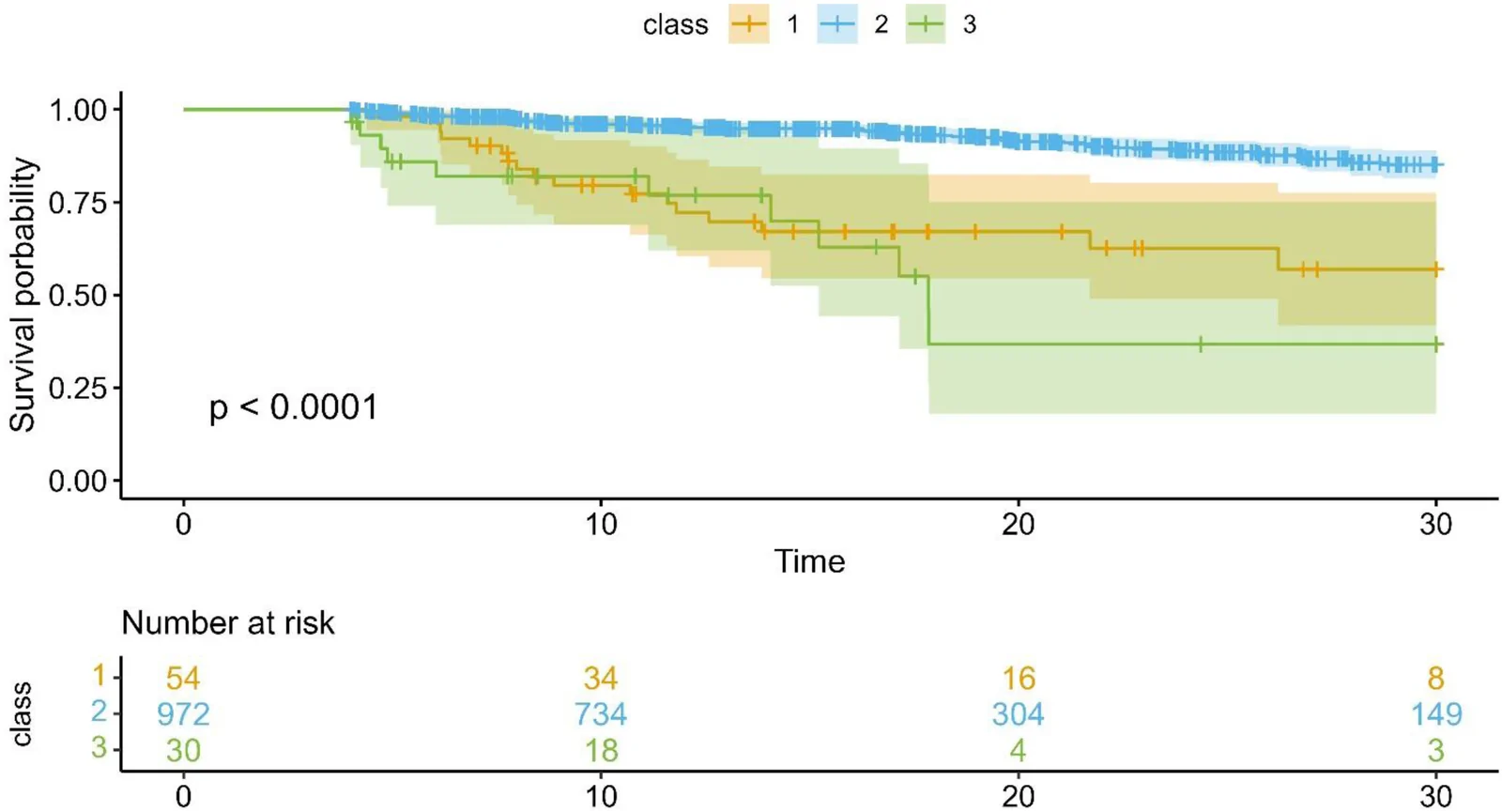
Kaplan-Meier analysis for 30-day survival probability in pediatric AKI patients.

A multivariable Cox regression model was employed to assess the association between serum lactate and 30-day mortality in pediatric AKI patients (Table 3). Initial multicollinearity diagnostics revealed VIF > 5 for several covariates (PLT, ALT, AST and PCT). Thus, PLT, ALT and PCT were excluded from the final model. After multivariable adjustment, a higher admission serum lactate was significantly associated with increased all-cause mortality at 30 days (HR 1.13, 95% CI 1.08–1.18, *p* < 0.01). Restricted cubic spline analysis demonstrated a monotonically increasing risk of 30-day mortality with rising admission serum lactate levels in children with AKI (overall *p* < 0.01; *p* for non-linearity = 0.46), as shown in Figure 3. In a multivariable Cox regression model adjusted for potential confounders and with the low stable group (Class 2) as the reference, both the high decreasing and persistent high trajectories were associated with a significantly increased risk of 30-day mortality. The hazard ratio for Class 1 was 3.37 (95% CI 1.83–6.24, p < 0.01), while Class 3 demonstrated the highest risk, with a hazard ratio of 6.24 (95% CI: 2.95–13.17, p < 0.01). The proportional hazards assumption was assessed using Schoenfeld residual global tests, which confirmed that the assumption was not violated for either model (admission lactate model: global p = 0.21; trajectory class model: global p = 0.14). These findings were further supported by Kaplan-Meier survival analysis, which demonstrated a statistically significant divergence in survival probabilities among the three groups (Log-rank p < 0.01). The persistent high group (Class 3) exhibited the poorest survival, followed by the high decreasing group (Class 1), while the low stable group (Class 2) had the most favorable outcome (Figure 4).

**Table 3:** Multivariable Cox regression for outcome in pediatric patients with AKI.

Variables	Crude Model HR (95% CI)	p-Value	Model 1 HR (95% CI)	p-Value	Model 2 HR (95% CI)	p-Value
Serum lactate	1.14 (1.10, 1.18)	<0.01	1.14 (1.10, 1.18)	<0.01	1.13 (1.08, 1.18)	<0.01
Serum lactate trajectory						
Class 1	4.68 (2.76, 7.95)	<0.01	4.69 (2.76, 7.97)	<0.01	3.37 (1.83, 6.24)	<0.01
Class 2	Reference		Reference		Reference	
Class 3	7.20 (3.81, 13.60)	<0.01	6.76 (3.56, 12.84)	<0.01	6.24 (2.95, 13.17)	<0.01

Model 1: unadjusted.

Model 2: Adjusted for age, gender.

Model 3: Adjusted for age, gender, sepsis, shock, malignant tumor, hepatic dysfunction, temperature, heart rate, respiratory rate, RBC, WBC, hemoglobin, CRP, creatinine, BUN, albumin, potassium, sodium, CKMB, AST, pH, APTT, INR, PT, Vasoactive drug.

Abbreviations: AKI, acute kidney injury; RBC, red blood cell; WBC, white blood cell; CRP, C-reactive protein; CKMB, creatine kinase-MB isoenzyme; AST, aspartate aminotransferase; APTT, activated partial thromboplastin time; INR, international normalized ratio; PT, prothrombin time; BUN, blood urea nitrogen; HR, hazard ratio; CI, confidence interval.

## Discussion

The present study investigated for the first time the association between both admission lactate levels and early lactate trajectories with 30-day all-cause mortality in pediatric patients with AKI. The main findings are as follows: First, admission lactate levels were monotonically associated with an increased risk of 30-day mortality. Second, using LGMM based on daily serum lactate measurements collected during the first four days of PICU admission, three distinct serum lactate trajectories were identified: Class 1 (high decreasing group), Class 2 (low stable group), and Class 3 (persistent high group). Notably, these trajectories demonstrated significant prognostic differences, with Class 3 showing the poorest clinical outcomes.

Lactate is a commonly used biomarker reflecting tissue perfusion, oxidative metabolism, and cellular dysfunction. Its prognostic value is particularly notable in patients with AKI, as AKI impairs lactate clearance, while systemic inflammation and microcirculatory dysfunction further exacerbate tissue hypoxia [17, 18, 19]. Numerous studies have demonstrated that elevated blood lactate levels are significantly associated with short-term mortality in AKI patients [12,13]. However, most existing studies have relied on lactate measurements at a single time point, which limits their ability to capture the dynamic evolution of lactate levels in response to disease progression and therapeutic interventions. In critically ill patients, the clinical course is continuously evolving, and treatments such as fluid resuscitation, vasoactive agents, and renal replacement therapy can all influence lactate levels. In recent years, dynamic lactate trajectory analysis has emerged as a valuable approach in critical care research. To date, relevant studies have been limited to adult populations. Children differ substantially from adults in terms of metabolic rate, hepatic and renal functional reserve, etiology, and treatment response, and findings from adult studies cannot be directly extrapolated to pediatric populations. Moreover, no study has systematically investigated the association between dynamic lactate trajectories and short-term outcomes in children with AKI.

The findings of this study confirm previous observations that elevated admission lactate levels are closely associated with adverse clinical outcomes. It should be emphasized that elevated lactate itself is not a direct cause of death, but rather an important biomarker of critical illness. The association between hyperlactatemia and increased mortality involves multiple pathophysiological mechanisms. First, tissue hypoperfusion and hypoxia represent the core mechanisms driving lactate elevation. Under hypoxic conditions, cells shift from aerobic ATP production to anaerobic glycolysis, leading to the conversion of pyruvate to lactate [20]. This disruption of cellular energy metabolism impairs cell function, affecting myocardial contractility and neuronal activity, thereby increasing the risk of multiple organ dysfunction [21,22]. Second, mitochondrial dysfunction plays a critical role in lactate accumulation. Impaired entry of pyruvate into the mitochondria for aerobic oxidation results in lactate buildup [23]. Moreover, mitochondrial dysfunction can induce apoptosis and pyroptosis, exacerbating endothelial injury and organ failure [24]. Third, elevated lactate induces metabolic acidosis. Increased hydrogen ion concentration inhibits various enzymes and interferes with cellular metabolism and ion channel function, leading to altered myocardial excitability and reduced vascular responsiveness to vasoactive agents, thereby worsening circulatory failure [25]. In addition, impaired lactate clearance contributes to persistent hyperlactatemia. The liver and kidneys are the primary organs responsible for lactate metabolism. Hepatic or renal dysfunction, as well as impaired cellular lactate uptake associated with sepsis, can reduce clearance capacity. Lactate accumulation further aggravates acidosis and cellular dysfunction, establishing a vicious cycle. Furthermore, hyperlactatemia activates inflammatory signaling pathways, promoting the release of pro-inflammatory cytokines such as IL-6 and TNF-α. This can induce or exacerbate systemic inflammation, leading to immune dysregulation, increased risk of infection, and aggravated organ damage [26,27]. Collectively, these interconnected mechanisms form a vicious cycle that ultimately contributes to a significantly increased risk of death.

Utilizing latent growth mixture model, the present study identified three distinct lactate trajectories. Class 2 maintained lactate levels within the near-normal range throughout PICU admission and exhibited the lowest mortality. In contrast, both Class 1 and Class 3 were associated with significantly higher 30-day mortality than Class 2, with Class 3 showing the poorest survival, indicating that a persistently elevated lactate trajectory represents the subgroup with the worst prognosis among pediatric AKI patients. Notably, although Class 1 and Class 3 both presented with elevated lactate levels, they differed substantially in clinical characteristics, underlying pathophysiology, and outcomes. As shown in Table 1, patients in Class 1 had higher admission lactate levels, more severe acidosis, and worse hepatic, renal, and coagulation parameters, suggesting a more critical condition at baseline with severe tissue hypoperfusion and multi-organ impairment. In Class 1, despite markedly elevated admission lactate, lactate levels declined rapidly during subsequent treatment, indicating a favorable response to early resuscitative measures, with prompt improvement in tissue perfusion and restoration of lactate clearance capacity. Thus, Class 1 represents reversible hyperlactatemia. By contrast, patients in Class 3 did not have the highest admission lactate levels, but their lactate levels continued to rise during the PICU stay without effective decline. This trajectory pattern suggests treatment-resistant hyperlactatemia, in which standard therapeutic interventions are insufficient to reverse the underlying pathophysiology, ultimately leading to the poorest outcomes. From a clinical perspective, these findings have important implications for risk stratification and management. For Class 1 (high decreasing group), despite critical illness at admission, early aggressive intervention may effectively reverse the clinical course. Thus, adequate resuscitative efforts and close monitoring of lactate clearance are warranted to assess treatment response. For Class 3 (persistent high group), conventional therapy may prove inadequate, and escalation of treatment, such as earlier initiation of renal replacement therapy or even extracorporeal membrane oxygenation, should be considered. For Class 2 (low stable group), a less intensive monitoring strategy may be appropriate to avoid unnecessary interventions.

The present study has several strengths. First, it represents the first investigation to identify dynamic lactate trajectories in pediatric patients with AKI using latent growth mixture model based on daily lactate measurements over the first four days of PICU admission, thereby addressing a significant gap in the literature for this population. Second, in contrast to traditional single-point lactate measurements, dynamic trajectory analysis distinguishes reversible hyperlactatemia from treatment-resistant hyperlactatemia, providing insights into treatment responsiveness and offering more refined information for clinical risk stratification. Nevertheless, several limitations should be acknowledged. First, the data were derived from a single center, and the generalizability of the findings requires validation through multicenter prospective studies. Second, Class 3 (persistent high group) accounted for only 2.84% of the total cohort, and the relatively small sample size limits statistical power. Third, detailed information regarding the timing and dosage of therapeutic interventions, such as fluid resuscitation and renal replacement therapy, was not recorded, precluding analysis of their direct impact on lactate trajectories. Fourth, patients who died within the first four days of PICU admission were not included, which may introduce survival bias and affect the completeness of trajectory classification. Fifth, urine output data in this retrospective database had inherent limitations, including potential inaccuracies in infants using diapers and interference from diuretic therapy, which may have introduced bias in AKI staging.

## Conclusions

The present study demonstrates that admission lactate levels in pediatric patients with AKI are significantly and positively associated with 30-day mortality. Compared with traditional single-point lactate measurement, lactate trajectory analysis not only identifies patient subgroups with distinct prognoses but also provides insights into treatment responsiveness.

## Authors’ contributions

Conceptualization, methodology, data curation and writing-original draft were performed by Xiaodong Jiang. Formal analysis and writing-original draft were performed by Yu Zhang. Methodology and writing-review and editing was performed by Hua Lu.

## Ethics approval and consent to participate

This study involves human participants and this project has been approved by the Institutional Review Committee of the Children’s Hospital of Zhejiang University School of Medicine (ethics reference number: 2019-IRB-052), and all procedures were conducted in accordance with the ethical standards of the Declaration of Helsinki and relevant regulations. Given the retrospective nature of the study and the use of anonymized data, the requirement for informed consent was waived.

## Declaration of generative AI

No generative AI tools were used in the preparation of this manuscript.

## Funding

The author(s) declare that no financial support was received for the research, authorship, and/or publication of this article.

## Data availability

The datasets presented in this study can be found in online repository: https://www.physionet.org/content/picdb/1.1.0/.

## Conflicts of interest

The authors declare that the research was conducted in the absence of any commercial or financial relationships that could be construed as a potential conflict of interest.
